# Supplementary material for: Study of Asian indexes by a newly derived dynamic model
Source: PLoS One. 2022 May 2;17(5):e0266600. doi: 10.1371/journal.pone.0266600 (PMC9060367; doi:10.1371/journal.pone.0266600)
Supplement: S1 Appendix — (PDF) [file pone.0266600.s001.pdf]

The martingale approach

Based on the theory of efficient markets, the market prices fully reflect available information, and can be expressed as

$$E\left(\frac{S(t+1)-S(t)}{S(t)}\middle|\Phi(t-1)\right)=E\left(\frac{S(t+1)-S(t)}{S(t)}\right)\quad almost\quad surely,$$

where E is the expectation operator, $S(t)$ is the stock price at time t, $\Phi(t-1)=\{S(t-1),S(t-2),\cdots\}$ is a general symbol for the set of information which is assumed to be fully reflected in the price at t.

Making τ-step forecasts based on the martingale yields the expected stock prices $\hat{S}(t+i)_{Mar}=S(t)$ for $i=1,\cdots,\tau$. The expected stock prices remain constant for τ days in the future owing to the nature of the martingale. Consequently, the martingale does not offer a trend prediction for the stock prices.

In this study, we set the martingale as the baseline which gives the best guess of the next price when the market is efficient. However, with the presence of market inefficiency due to reasons such as information asymmetries, the market prices will not be the martingale sequence. On the condition, the predictability of stock market prices exists.
