# Supplementary material for: Study of Asian indexes by a newly derived dynamic model
Source: PLoS One. 2022 May 2;17(5):e0266600. doi: 10.1371/journal.pone.0266600 (PMC9060367; doi:10.1371/journal.pone.0266600)
Supplement: S2 Appendix — (PDF) [file pone.0266600.s002.pdf]

The curve fitting approach

We employ the cubic curve fitting to expresses the stock movements as a cubic function in a specified period/window. The coefficients of the cubic function are constant in the period, and change with time as the window rolls forward.

We specify a period of 200 trading days as a window, and the stock prices can be represented as

$$S(t) = \alpha t^3 + \beta t^2 + \gamma t + \delta, \quad t \in [t_1, t_{200}],$$

where α, β, γ, δ are estimated by ordinary least squares method, and denoted as $\hat{\alpha}$, $\hat{\beta}$, $\hat{\gamma}$, $\hat{\delta}$. The τ-step forecasts are then made as $\hat{S}(t+i)_{CF} = \hat{\alpha}(t+i)^3 + \hat{\beta}(t+i)^2 + \hat{\gamma}(t+i) + \hat{\delta}$ for $i = 1, \cdots, \tau$. We shift the window by one day at a time to obtain the next τ-step forecasts until we cover the entire data.
