# Supplementary material for: Study of Asian indexes by a newly derived dynamic model
Source: PLoS One. 2022 May 2;17(5):e0266600. doi: 10.1371/journal.pone.0266600 (PMC9060367; doi:10.1371/journal.pone.0266600)
Supplement: S1 Table — (PDF) [file pone.0266600.s003.pdf]

S3 Table Correct up-trend (CP) (%) for the testing set.

		$\tau = 1$	$\tau = 2$	$\tau = 3$	$\tau = 4$	$\tau = 5$
**Nikkei 225**	nPRM	54.92	54.95	55.66	60.29	58.51
	Curve fitting	52.00	51.50	51.99	57.14	55.99
**Hang Seng**	nPRM	52.92	48.52	50.32	49.18	50.16
	Curve fitting	52.88	50.00	50.84	51.86	55.44
**TAIEX**	nPRM	54.68	52.98	57.31	58.26	59.66
	Curve fitting	55.79	59.03	59.26	61.39	61.69
**KOPSI**	nPRM	49.82	52.16	51.69	52.16	55.29
	Curve fitting	52.78	51.03	52.08	50.53	48.39
**PSEi**	nPRM	49.24	50.74	50.55	49.81	48.18
	Curve fitting	45.79	45.79	47.89	45.79	45.55
**SET**	nPRM	49.42	49.41	47.89	51.91	49.80
	Curve fitting	50.57	48.26	49.12	52.05	51.74
**BSE SENSEX**	nPRM	48.07	51.85	53.67	57.01	59.32
	Curve fitting	52.68	55.79	58.44	59.83	64.17
**STI**	nPRM	46.04	49.46	50.90	54.06	54.74
	Curve fitting	50.25	52.43	50.95	54.21	55.50
**JKSE**	nPRM	51.93	57.21	55.28	55.69	56.11
	Curve fitting	51.09	53.01	51.35	52.66	51.83
**KLCI**	nPRM	46.89	45.53	42.17	44.53	48.51
	Curve fitting	50.36	49.25	43.51	46.99	45.32
