# Supplementary material for: Study of Asian indexes by a newly derived dynamic model
Source: PLoS One. 2022 May 2;17(5):e0266600. doi: 10.1371/journal.pone.0266600 (PMC9060367; doi:10.1371/journal.pone.0266600)
Supplement: S2 Table — (PDF) [file pone.0266600.s004.pdf]

S4 Table Correct down-trend (CD) (%) for the testing set.

		$\tau = 1$	$\tau = 2$	$\tau = 3$	$\tau = 4$	$\tau = 5$
**Nikkei 225**	nPRM	44.71	45.02	43.19	38.11	39.76
	Curve fitting	42.01	41.46	39.51	35.36	37.99
**Hang Seng**	nPRM	45.27	45.74	46.13	44.33	41.79
	Curve fitting	44.75	46.94	46.74	47.46	47.97
**TAIEX**	nPRM	44.30	41.35	42.00	42.08	41.63
	Curve fitting	45.54	48.00	44.67	45.96	44.29
**KOPSI**	nPRM	44.63	46.10	47.24	45.96	50.51
	Curve fitting	47.18	45.12	47.84	45.10	44.52
**PSEi**	nPRM	51.15	51.57	46.25	48.63	48.00
	Curve fitting	48.35	49.45	42.31	45.05	46.41
**SET**	nPRM	48.26	48.67	49.42	50.00	48.33
	Curve fitting	48.55	47.43	50.57	49.43	50.29
**BSE SENSEX**	nPRM	42.33	38.02	37.27	42.44	39.34
	Curve fitting	41.83	35.42	34.25	37.06	36.50
**STI**	nPRM	47.79	48.46	48.08	49.21	49.60
	Curve fitting	51.67	49.71	47.37	47.90	49.08
**JKSE**	nPRM	47.40	47.44	48.55	44.94	45.38
	Curve fitting	43.01	40.64	38.38	36.81	33.52
**KLCI**	nPRM	51.22	48.09	50.00	50.74	52.46
	Curve fitting	49.03	46.42	46.13	46.07	42.11
