# Supplementary material for: Study of Asian indexes by a newly derived dynamic model
Source: PLoS One. 2022 May 2;17(5):e0266600. doi: 10.1371/journal.pone.0266600 (PMC9060367; doi:10.1371/journal.pone.0266600)
Supplement: Attachment — Submitted filename: Response to Reviewers.docx [file pone.0266600.s005.docx]

Submission No: PONE-D-21-10180

Dear Editor,

We appreciate the reviewers’ valuable comments and suggestions. Meanwhile, we have addressed those comments one by one in the following responses. In the following, the original comments from the reviewer are in **black** and our responses are in **blue**.

With Regards,

Yong Shiuan, Lee

=======================================================

**Response to Comments from Reviewer #1**

**1. Format of the paper is weak, please correct.**

We rearrange the outline of this article. We follow the submission guidelines of the “Manuscript Organization” of PLOS ONE. We present the sections/subsections as follows.

•Abstract

•Introduction

•Methods

∙ The Price Reversion Model (PRM)

∙ Derivation of solutions to the new Price Reversion Model (nPRM)

∙ Implementation details of the nPRM

∙ Model evaluation

•Results

∙ Simulation design

∙ Applied data

∙ Simulation results

•Discussion

•Acknowledgments

•References

•Supporting information captions

∙ S1 Appendix - The martingale approach

∙ S2 Appendix - The curve fitting approach

∙ S3 Fig.

∙ S4 Fig.

∙ S5 Fig.

∙ S6 Fig.

**2. Include the contribution of the paper in the abstract, Introduction and Conclusion.**

We rewrite the Abstract, the Introduction Section, and the Conclusion Section. The distinguishing features and the contribution of the proposed model are presented in the end of the Introduction Section. The contribution of our work is included in the Abstract, the Introduction Section, and the Conclusion Section.

In details:

Abstract (the last two sentences):

“This proposed procedure brought a different perspective to the study of stock prices. The time varying coefficients in the nPRM offer economic meanings of the stock movements.”

Introduction Section (line 90-97):

“The contributions of this paper are in the following.

The proposed procedure of nPRM directly analyzes raw data of stock prices, which retains all available information up to date to avoid reducing low-frequency information by taking returns or the logarithm of returns as well as making assumptions about the model. Moreover, the coefficients in the nPRM, namely the equilibrium price and the gradient, have their very own economic meanings. The gradient value reflects the convergence speed to the equilibrium price. We may further utilize the series of the gradient values as a feature for other models.”

Discussion Section (line 382-386):

“From the viewpoint of signal processing, the proposed procedure of nPRM models the stock prices with all available information. Therefore, the coefficients in the nPRM preserve and reveal more information about the stock movements economically. They also may be treated as important features for forecasting by advanced models such as deep learning models.”

**3. The is no background used at all. A method focused paper is not interesting for the investors. Please add a background on the stock market index forecasting and take them out of the Introduction.**

We add some background of stock market. We also rewrite the Introduction Section to review approaches of stock forecasting from different perspectives, such as financial accounting, econometrics, signal processing plus artificial intelligence models, differential equations and dynamic systems.

In details:

Introduction Section (line 3-68):

“The market prices of an individual stock reflect the company's existing value or future profit growth. A stock index, measured by the weighted mean prices of selected stocks in a stock market, represents the stock market's performance and potential competitive ability. Investors who have different motives for participating in the market concern about the changes of a stock or a stock index's market prices of different scales, intraday high-frequency data, daily closing prices, weekly or monthly summary, and etc. Accurately forecasting the stock movements could help the investors with decisions about investment strategy.

Whether the stock prices can be forecasted has been repeatedly discussed in finance. The efficient market hypothesis forms a foundation of the theory that under what circumstances the stock prices is predictable. The efficient market hypothesis states that three types of markets can be distinguished as the weak form, the semi-strong form, and the strong form depending on the level of market efficiency. The stock prices fully reflect all available market information in a strong efficient market, thus they are not predictable. The martingale depicts the stock movements in the strong efficient markets by the theory of random walks. In contrast, many real markets are considered as the weak or the semi-strong efficient markets. Consequently, to forecast the prices in these markets is possible.

The analysis of the stock movements is based on either the stock prices or the stock returns. The choice between the stock prices and the returns mainly depends on the approach adopted. …”

**4. Please clearly indicate why you needed the PRM method instead of any other forecasting method like Gradient Boosting.**

The gradient boosting is an ensemble method which combines weak prediction models (classification, regression, etc.) to form a predictive model. This is a well known and commonly used technique in machine learning. We are really grateful for this valuable comment although we did not involve this in the previous manuscript. On the contrary, our proposed model takes a different perspective by using the differential equations and the dynamic systems. We choose the martingale as a baseline/benchmark to compare with the nPRM if the market is efficient. Additionally, we choose the curve fitting to compare with the nPRM since in our proposed procedure the coefficients are determined piecewisely (constant in each window). The coefficients change with time (window by window). Therefore, the dynamics of the coefficients characterizes the stock movements. This procedure is similar to the approach of curve fitting, which solves the parameters by OLS piewisely. Those parameters also change in different intervals. Since the PRM and the nPRM are deterministic models, which solve the differential equations in an interval [t_0_, t_1_], it is hard for us to apply the gradient boosting during our proposed procedure or compare the nPRM with it. However, we think that the gradient boosting may be implemented to find the best values of the coefficients (κ and *A*) in the nPRM in the future. Comparing the results of the nPRM and a boosted predictive model such as combining SVRs (support vector regression) may also be the future work.

**5. Number of references has to be at least 30.**

We add some references as follows. Cowles (1933), Abarbanell and Bushee (1997, 1998), Park and Irwin (2007), Malkiel and Fama (1970), Campbell and Shiller (1988 [9]; 1988 [12]; 2005), Lettau and Ludvigson (2001), Lewellen (2004), Cochrane (2008; 2011), Welch and Goyal (2008), Rapach et al. (2010), Cheng and Wei (2014), Lim and Luo (2012), Wang and Wang (2015), Zhang and Pan (2015), Yan et al. (2020), Basak et al. (2019), Xu et al. (2016), Mallikarjuna and Rao (2019). We also brief important references in the Related Work Section.

In details:

The number of references is 38 now.

=======================================================

**Response to Comments from Reviewer #2**

**1. In the recent decades, the literature on asset pricing has been prolific in providing us with new predictors that have increased substantially the predictive power of return forecasting models, especially in the case of stock market indexes. Thus, the classic view of financial market, where returns were almost unforecastable –consistent with the efficient-market hypothesis (Fama, 1970)–, has been clearly overcome by different models that exploit the strong predictive power of slow-moving predictors, such as the dividend yield, the price-to-earnings ratio, or the consumption-wealth ratio. Although this paper adopts a completely different perspective to analyze the stochastic behavior of stock indexes, I believe that the authors should better place their contribution in the current body of work, referring to important papers, such as Campbell and Shiller (1988ab, 2005), Lettau and Ludvigson (2001), Lewellen (2004) or Cochrane (2008). Cochrane (2011) provides a superb revision on the topic.**

We add some references as follows. Cowles (1933), Abarbanell and Bushee (1997, 1998), Park and Irwin (2007), Malkiel and Fama (1970), Campbell and Shiller (1988 [9]; 1988 [12]; 2005), Lettau and Ludvigson (2001), Lewellen (2004), Cochrane (2008; 2011), Welch and Goyal (2008), Rapach et al. (2010), Cheng and Wei (2014), Lim and Luo (2012), Wang and Wang (2015), Zhang and Pan (2015), Yan et al. (2020), Basak et al. (2019), Xu et al. (2016), Mallikarjuna and Rao (2019). We also brief important references in the Introduction Section.

In details:

Introduction Section (line 11-80) (partly):

“Whether the stock prices can be forecasted has been repeatedly discussed in finance (Cowles1933, Lim2012). The efficient market hypothesis (Malkiel1970) forms a foundation of the theory that under what circumstances the stock prices is predictable.”,

“In contrast, many real markets are considered as the weak or the semi-strong efficient markets (Cochrane2011, Lim2012).”,

“The analysis of the stock movements is based on either the stock prices or the stock returns. The choice between the stock prices and the returns mainly depends on the approach adopted. From the financial accounting perspective, fundamental analysis (Abarbanell1997, Abarbanell1998) and technical analysis (Park2007) are the two main intellectual traditions.”,

“When using ARIMA and VAR models, the stationarity of the time series is required. Therefore, the stock returns instead of the stock prices are the primary research subject (Campbell1988b, Lewellen2004, Welch2008). Financial ratios like the log dividend-price ratio (Campbell1988[9], Campbell2005), the dividends (Campbell1988[12]), the price–earnings ratio (Campbell2005, Lewellen2004), the dividend yield (Cochrane2008, Lewellen2004), the consumption-wealth ratio (Lettau2001), the book-to-market ratio (Lewellen2004) serve as important predictors for asset returns in regression models, particularly VAR models. Although some good predictors in financial literature have restricted predictive ability for the equity premium due to model uncertainty and instability (Welch2008), combining individual forecasts produce out-of-sample gains (Rapach2010). Modeling stock returns reduces low-frequency information, the trend for example, from the perspective of signal processing. Therefore, forecasting the stock prices based on models constructed from detrended data may be less accurate in this sense.”,

“The artificial intelligence models applied to the stock prices in existing studies include support vector regression (SVR) (Cheng2014), neural networks (Atsalakis2009, Guresen2011, Wang2015, Zhang2015, Yan2020), tree-based models (Basak2019), etc. To applying the artificial intelligence models, acquiring features is an important step. The empirical mode decomposition (EMD) (Cheng2014, Zhang2015), the ensemble empirical mode decomposition (EEMD) (Xu2016), and the complementary ensemble empirical mode decomposition (CEEMD) (Yan2020) are widely employed methods to decompose the stock prices, which are regarded as signals. The empirical results of US and UK stock indexes show that the artificial intelligence models are better than the ARIMA and GARCH models in terms of forecasting directional symmetry (Zhang2015). Furthermore, the CEEMD-PCA-LSTM model (Yan2020), which integrate the CEEMD, principal component analysis (PCA), and long short-term memory (LSTM) networks, outperforms naive recurrent neural networks (RNN) or LSTM networks in terms of forecasting accuracy for six selected stock indexes. Still, in a review study of the returns from financial perspective (Mallikarjuna2019), the empirical evidences show that no single model out of statistical, artificial intelligence, frequency domain, and hybrid models under investigation could be applied uniformly to all markets. On the other hand, the direction prediction problem is another task, which can be tackled by the tree-based models (Basak2019) to predict the stock price direction.”,

“Another study (Xu2016) also considers the stock markets as complex dynamical systems, but their interactions are analyzed by methods of signal processing, the EMD and the EEMD along with detrended cross-correlation analysis (DCCA).”,

**2. I believe that the main contribution of the paper and the usefulness of the proposed model should be better explained in the Introduction Section. Although the paper is clear and concise, in my opinion these aspects have not been sufficiently addressed.**

We rewrite the Introduction Section. The distinguishing features and the contribution of the proposed model are presented in the end of the Introduction Section.

In details:

Introduction Section (line 90-97):

“The contributions of this paper are in the following.

The proposed procedure of nPRM directly analyzes raw data of stock prices, which retains all available information up to date to avoid reducing low-frequency information by taking returns or the logarithm of returns as well as making assumptions about the model. Moreover, the coefficients in the nPRM, namely the equilibrium price and the gradient, have their very own economic meanings. The gradient value reflects the convergence speed to the equilibrium price. We may further utilize the series of the gradient values as a feature for other models.”

**3. In line with the previous comment, I believe that the explanations provided in the Results Section are excessively synthetical. The authors should make an effort to better describe the results and their implications, which is somewhat applicable to the Conclusions Section.**

We amend the Results Section with some more discussions. We also rewrite the Discussion Section (Conclusion Section in previous version of manuscript) to highlight the contribution of this article and point out possible future work.

In details:

Result Section (line 308-372) (important part):

“From the deviation-type errors, the martingale (see details in S1 Appendix) has the smallest errors followed by those from the nPRM, the PRM and the curve fitting (see details in S2 Appendix) in the ascending order. Comparing the nPRM with the PRM, although the PRM obtains smaller 1-step forecast errors, the PRM obtains larger errors than the nPRM with the increase of the number of forecasting steps. From the overall performance, the nPRM is more accurate than the PRM. The PRM seems to perform better at the 1-step forecast since the miscalculations for cases 3 and 4 of the nPRM in Table 1 do not lead to large errors. However, with the increases of the number of forecasting steps, those miscalculations of theoretical values accumulate errors.”

“The trend-type errors cannot be applied to the forecasts of the martingale since the martingale forecasts the price of the next day by the present price. Therefore, we compare the trend-type errors between the other three methods. The PRM, nPRM, and the curve fitting have similar percentage of correct trend forecasts. Although the differences between the three methods are not significant, the PRM produces slightly higher trend accuracy among these three methods.”

“We summarize the performances of the methods as follows. Considering the deviation-type forecasting errors, the martingale seems to perform the best among the methods. According to the empirical evidence (Lim2012), the test based on the martingale can be used to examine the market efficiency of the stock market. Despite the absence of the test in this study, the low errors of the martingale for the four Asian stock indexes imply that the markets tend to be efficient during the period of the data. The martingale suggests that no information we can obtain from the past to forecast the future stock prices. Under the circumstances, our proposed method nPRM captures the trend of the stock movements quite well, and provides meaningful coefficients, *A* and κ, as well. If applying the nPRM to the markets that are less efficient, the model coefficients may acquire more information with economic meanings and obtain more accurate forecasts than the martingale.

The PRM has some flaw in the integration process and does not require smoothing for the stock prices. These disadvantages cause errors in forecasts. The errors are small when the time interval of forecast is short, but they grow with the increase of the time length. The newly proposed method nPRM improves these weaknesses. Therefore, the forecasting errors are notably smaller than the PRM for multiple step forecasts.

By the deviation-type measurements, the PRM or nPRM outperforms the curve fitting while the martingale cannot deliver a forecast of trend.

In spite of small differences, the PRM has the highest forecasting accuracy in all of the four Asian markets, and produces better directional forecasts for the up trend with higher CP values when the nPRM has higher CD values. In brief, the trend forecasting ability of the three methods are close.”

Discussion Section (line 387-399):

“We evaluate the forecasting accuracy of the nPRM along with the PRM, the martingale, and the cubic curve fitting by four Asian stock index examples. By investigating the daily closing prices, the nPRM accurately characterizes and forecasts the large-scale motions in these four stock markets. Although the forecasting accuracy declines over time, which is the same phenomenon for all the methods, the deviation-type errors of the nPRM are the smallest except for the martingale. However, the martingale can not be employed to forecast the trend of the stock even though it offers an eligible prediction about the prices if the market is efficient.

The trend-type errors of the nPRM, the PRM, and the curve fitting are close. This indicates that the trend forecasting ability of the nPRM does not outperform the other methods as its overall forecasting ability. In order to improve the trend forecasting in the future, we will develop a more sophisticated method to calculate the κ values without the gradient control.”

4. **Although the manuscript is well-written, there are some typos that should be corrected (e.g., “the primary goals in this study is to identify” in line 36, etc.).**

We corrected the typo in line 86 (line 36 in the previous version).

🡺We changed the “is” to “are”.

All the expressions, “stock movement”, are changed as “stock movements” for consistency throughout the paper (three times in the Abstract and in line 9, 17, 21, 31, 80, 205, 248, 358, 385).

All the expressions, “stock price”, are changed as “stock prices” for consistency throughout the paper.

We corrected the typos for the solutions of the PRM in line 126-133.

🡺Cases A, B, C, D and equations (2), (3), (4), (5).

We corrected the typos for the solutions of the nPRM.

🡺 S_0_ < A (line 162), S_0_ > A (line 166), S_0_ < A (line 170), increasing (line 171), S(t) < A (line 171), S_0_ > A (line 174), decreasing (line 175), S(t) > A (line 176).

**5. TAIEX is first mentioned in the paper in line 16, while the acronym is defined in 21. It is important that the authors correct these minor issues throughout the document.**

The acronym is defined when first mentioning TAIEX in line 74 (in the revised version).

=======================================================

**Response to Comments from Reviewer #3**

**1. Newly derived Price reversion model (nPRM) may be a revised version of original Price reversion model (PRM). As the background of this study, PRM should be introduced and nPRM is compared with PRM in order to reveal their difference.**

We add a subsection to describe the original Price Reversion Model (PRM) in the Methods Section. We also add Table 1 to compare the solutions of the PRM with the solutions of the nPRM.

In details:

Methods Section – The Price Reversion Model (PRM) subsection (line 122-136)

Methods Section - Derivation of solutions to the new Price Reversion Model (nPRM) (line 177-184)

“A comparison of the cases on different conditions between PRM and nPRM is given by Table 1. As mentioned before, cases B and D in the PRM do not exist due to zero external force at *A*. By examining the phase plots of Figure 1 and Figure 2, the case A of the PRM can be further divided into cases 2 and 4 of the nPRM. The case C of the PRM can be further divided into cases 1 and 3 of the nPRM. We derive solutions for the cases 3 and 4 of the nPRM, which can not be obtained identically from the solutions of the PRM. As a result, the theoretical values obtained via the PRM are not so accurate as those from the nPRM.”

**2. Martingale and Curve fitting are also described briefly because nPRM is compared with them.**

We add Appendix S1 and S2 to describe the Martingale and the Curve fitting.

In details:

We mention S1 Appendix and S2 Appendix in line 316 and 317.

**3. Numerical examples reveal that PRM is better than nPRM at small value of tau and that Martingale seems to be best among them. The validity of nPRM is not obvious. Authors should discuss these points.**

We compare the solutions of the PRM with the solutions of the nPRM by Table 1. We also add some paragraphs of discussion in the last part of the Results Section.

In details:

Derivation of solutions to the new Price Reversion Model (nPRM) Section (line 177-184):

“A comparison of the cases on different conditions between PRM and nPRM is given by Table 1. As mentioned before, cases B and D in the PRM do not exist due to zero external force at $A$. By examining the phase plots of Figure 1 and Figure 2, the case A of the PRM can be further divided into cases 2 and 4 of the nPRM. The case C of the PRM can be further divided into cases 1 and 3 of the nPRM. We derive solutions for the cases 3 and 4 of the nPRM, which can not be obtained identically from the solutions of the PRM. As a result, the theoretical values obtained via the PRM are not so accurate as those from the nPRM.”

Results Section (line 317-324):

“Comparing the nPRM with the PRM, although the PRM obtains smaller 1-step forecast errors, the PRM obtains larger errors than the nPRM with the increase of the number of forecasting steps. From the overall performance, the nPRM is more accurate than the PRM. The PRM seems to perform better at the 1-step forecast since the miscalculations for cases 3 and 4 of the nPRM in Table 1 do not lead to large errors. However, with the increases of the number of forecasting steps, those miscalculations of theoretical values accumulate errors.”

**4. Computational cost is also important point of view. The computational costs of four algorithms, nPRM, PRM, Martingale and Curve fitting, should be compared.**

We add Table 7 to present average computational time of the four methods in the empirical study. We discuss the comparison in the last paragraph in the Results Section.

In details:

Results Section (line 373-378):

“The average computational times of all the four methods are investigated through 100 simulations. The average CPU times are shown in Table 7. The martingale uses the least computational time while the curve fitting spends the most. The nPRM and the PRM take nearly the same time, which is approximately half of the time used by the curve fitting. Even so, the nPRM is computationally efficient in view of the relationship between the forecasting accuracy and the computational time.”
