# Supplementary material for: Study of Asian indexes by a newly derived dynamic model
Source: PLoS One. 2022 May 2;17(5):e0266600. doi: 10.1371/journal.pone.0266600 (PMC9060367; doi:10.1371/journal.pone.0266600)
Supplement: Attachment — Submitted filename: Response to Reviewers.docx [file pone.0266600.s006.docx]

Submission No: PONE-D-21-10180

Dear Editor,

We appreciate the reviewers’ valuable comments and suggestions. Meanwhile, we have addressed those comments one by one in the following responses. In the following, the original comments from the reviewer are in **black** and our responses are in **blue**.

With Regards,

Yong Shiuan, Lee

===========================================================

**Response to Comments from Reviewer #3**

**1. The manuscript is revised according to the reviewer's comment.
However, the section titles should be numbered adequately for readers' convenience.**

We agree with this comment that numbered sections/subsections are more readable. As a matter of fact, we follow the submission guidelines of the “Manuscript Organization” of PLOS ONE, and organize the sections by using the downloaded sample file. The format of the template for LaTeX submission does not number the sections and subsections. Therefore, we cannot present the manuscript with numbered section titles.

We present the revised manuscript as follows.

•Abstract

•Introduction

•Methods

∙ The Price Reversion Model (PRM)

∙ Derivation of solutions to the new Price Reversion Model (nPRM)

∙ Implementation details of the nPRM

∙ Model evaluation

•Results

∙ Applied data

∙ Empirical results

•Discussion

•Acknowledgments

•References

•Supporting information captions

∙ S1 Appendix - The martingale approach

∙ S2 Appendix - The curve fitting approach

∙ S3 Table Correct up-trend (CP) (%) for the testing set.

∙ S4 Table Correct down-trend (CD) (%) for the testing set.

===========================================================

**Response to Comments from Reviewer #4**

**1. The abstract is too cumbersome and a bit long. Especially the first four sentences are somewhat redundant.**

We exclude the first four sentences in the abstract. We also amend and rearrange the sentences in the abstract since we add more empirical examples.

In details:

Abstract (from the 5^th^ line in the abstract):

“…This proposed procedure brought a different perspective to the study of stock prices based on thermodynamics, and the time varying coefficients in the nPRM offer economic meanings of the stock movements. More specifically, the average of smoothed historical data *A* in the nPRM, analogous to the environment temperature in the Newton's law of cooling, represent an implied equilibrium price. The heat transfer coefficient *k* is adapted to be either negative or positive, which illustrates the speed of convergence or divergence of stock prices, respectively. The empirical study of ten Asian stock indexes shows that the nPRM accurately characterizes and forecasts the market values.”

**2. In the introduction, the authors introduced a variety of methods for predicting stock prices, and commented that there is no single method that can be used in all markets. I think this evaluation is not objective. No method is universally applicable to all markets, including the method proposed in this article, which has poor predictive effect in efficient markets. Therefore, what are the differences between the nPRM and the existing methods, or what are the advantages of the nPRM in predicting prices? Please elaborate on the importance of proposing this method.**

In the previous edition of our manuscript (line 63-66), we summarize research results from reference [25] and state that “Still, in a review study of the returns from financial perspective [25], the empirical evidences show that no single model out of statistical, artificial intelligence, frequency domain, and hybrid models under investigation could be applied uniformly to all markets.” We would like to point out that disadvantage of modeling returns instead of stock prices is a loss of low-frequency information by the signal processing perspective. That’s why we choose to model daily closing prices in this work.

The proposed method nPRM produces predictions with relatively low Mean Absolute Percentage Errors (MAPEs) in our empirical study compared to other methods regardless of the market efficiency. More importantly, the difference in MAPE between nPRM and the martingale is little.

Indeed, “all models are wrong, but some are useful”, said by George Box. This quotation refers to not only statistical models but also other scientific models. However, we are dedicated to construct models that 1. have logical representation and economically reasonable explanations, and 2. produce predictions as accurately as possible.

The main contributions of this proposed method include describing the stock price movement accurately and provide economically meaningful coefficients at the mean time.

**3. The authors put forward two primary goals for PRM in the introduction. However, the contribution of PRM to price prediction does not seem to be expressed. Does PRM have a large impact on the academic field or is it worthy of being an improvement target? The second goal does not seem to have been achieved.**

The PRM is developed upon the Newton’s law of cooling, which is a well known thermodynamic model and introduced into finance in some researches, for example,

“Gkranas, A., Rendoumis, V.L. and Polatoglou, H.M. (2004). Athens and Lisbon stock markets – a thermodynamic approach, *WSEAS Transactions on Business and Economics*, Vol. 1, No. 1, pp.95–100”,

“Zarikas, V., Christopoulos, A.G. and Rendoumis, V.L. (2009). A thermodynamic description of the time evolution of a stock market index, *European Journal of Economics*, *Finance and Administrative Sciences*, Vol. 16, pp.73–83”,

“Todorović, J. Đ., Tomić, Z., Denić, N., Petković, D., Kojić, N., Petrović, J., & Petković, B. (2018). Applicability of Newton’s law of cooling in monetary economics. Physica A - Statistical Mechanics and its Applications, 494, 209-217”,

and our reference [27] among others.

Although PRM might not have brought great impact to the academic field of finance, the concept of introducing the thermodynamics into stock index dynamic modeling is new and draws some attention. This approach models the stock prices from a different perspective without loss of information due to transformation or model assumptions.

We slightly amend the introduction, especially the paragraph detailing the primary goals and contributions of this work (line 85-90).

In details:

Abstract (line 5-12):

“This proposed procedure brought a different perspective to the study of stock prices based on thermodynamics, and the time varying coefficients in the nPRM offer economic meanings of the stock movements. More specifically, the average of smoothed historical data *A* in the nPRM, analogous to the environment temperature in the Newton's law of cooling, represent an implied equilibrium price. The heat transfer coefficient *k* is adapted to be either negative or positive, which illustrates the speed of convergence or divergence of stock prices, respectively. The empirical study of ten examples shows that the nPRM accurately characterizes and forecasts the market values for Asian stock indexes.”

Introduction (line 85-94):

“Therefore, we identify the reasons of inaccurate forecasts by the PRM and propose to model the stock prices by a newly derived model, i.e. the new Price Reversion Model (nPRM). The contributions of this paper are in the following. The proposed procedure of nPRM directly analyzes raw data of stock prices, which retains all available information up to date to avoid reducing low-frequency information by taking returns or the logarithm of returns as well as making assumptions about the model. Moreover, the coefficients in the nPRM, namely the equilibrium price and the gradient, have their very own economic meanings. The gradient value reflects the convergence speed to the equilibrium price. We may further utilize the series of the gradient values as a feature for other models.”

**4. The first contribution cannot be considered a contribution. If you don't care about the prediction accuracy, all models can input raw data. But whether this is right is worth discussing.**

The first contribution is “directly analyzes raw data of stock prices, which retains all available information up to date to avoid reducing low-frequency information …” (line 87-90). Since a great part of recent researches of stock in academic field of finance is based on stock returns, we try to describe the stock price movement directly without loss of information from the signal processing perspective. We try to construct a reasonable model, which characterizes our subject properly and creates precise predictions.

We do care about the prediction accuracy, which is mainly measured by the Mean

Absolute Percentage Error (MAPE) in this article. Empirical study shows that the prediction accuracy of nPRM is really close to the martingale.

In the previous edition of our manuscript, the measurement trend accuracy (Acc) is used to determine whether the direction of the prediction is the same as real market value. This measurement along with CD and CP are useful in practice if we focus on the direction which stock prices go. For a martingale, either direction happens with a 50/50 chance. We replace Acc by another terminology, Directional Symmetry (DS), in our revised manuscript to avoid misunderstanding. Larger values of DS, CD, and CP represent more price predictions going in the same direction with real market.

In details:

Methods-Model evaluation (line 236-237, 244-249):

“directional Symmetry (DS)”,

“A smaller value of the MAPE indicates that the model better describes the movements of the stock prices. The MAE and the RMSE show similar tendency with the MAPE. DS measures the frequency of correct directional forecasts. CP and CD measure the frequency of correct directional forecasts when real market values rise and fall, respectively. The larger values of DS, CP, and CD suggest that the model obtains more correct directional forecasts in general, for uptrend, and for downtrend, respectively.”

**5. In the model part, what is the economic implications or intuition of nPRM? Why is the change in price assumed to be such a differentiable equation?**

The economic implication of nPRM is briefly described in the Section Derivation of solutions to the new Price Reversion Model (nPRM) (line 136-150). We refer *A* to the market equilibrium price. We refer *k* to the speed of convergence or divergence of stock prices, which is determined by the force on stock.

We assume that the dynamics of the price movement is differentiable (at least twice differentiable) to time *t*. However, in reality we observe the price at discrete timepoints. In this study, the observations are made once a day (daily closing prices). Since we cannot observe the price at an infinity of possible argument time *t*, we need smoothing technique to obtain differentiable curves that capture important trend and features of our data (step 1 in the implementation of nPRM). This smoothing step is neglected in PRM so that this modification in nPRM makes it more theoretically reasonable.

**6. The authors explain that nPRM is better than PRM because the former has two more cases than the latter. How do the authors identity it? Why is it not because smooth historical data is used when calculating A? Why is it not caused by controlling K?**

The solutions of nPRM are theoretically reasonable since they depict the behaviors of the dynamic system on 4 conditions separately. The old PRM solutions are not so clear, moreover, two cases (B and D) do not exist. The nPRM is better than PRM not only because it describes all the cases more clearly but also because the whole procedure applying nPRM is more sophisticated. For example, we add a smoothing step to eliminate the discrepancy between differentiable assumption and discrete observations. Also, the calculation of *k* is more polished than the original PRM.

We identify these solutions from the phase plane plots in Figures 1 and 2, which is commonly used when describing the behaviors of differential equations. These solutions are obtained theoretically by solving equation (1). During the solving process, the *A* and *k* are constant in the short time period *t* in [*t_0_*, *t_1_*]. Consequently, calculating *A* and controlling *k* are not supposed to affect the solutions in each period. However, when we dynamically model the stock prices with changing time periods, we need to consider the changes of *A* and *k*.

We further reply to the next comment (comment 7) for more details in estimating *A* and *k*.

**7. In nPRM, A and K are the most critical parameters, and their estimation method will directly determine the predicted performance. Authors should focus on how to estimate A and K. How to determine the optimal A and K is the key. From the prediction results, it is unreasonable to assume that the index obeys geometric Brownian motion.**

We think the assumption that stock index follows the geometric Brownian motion is reasonable. This is why that in the previous edition of manuscript we simulate sample paths of a stock index prices from the geometric Brownian in our Simulation Design Section (line 260-262 in the previous edition).

We re-assess the estimations of the coefficients, *M* (smoothing period) and *A* (equilibrium price), in this revision. In this revision, we determine the coefficients in the training dataset of 80% historical daily closing prices. As a result, we revise the Result Section and delete the Simulation design subsection.

In details:

Results (line 250-254):

“For application to real data, we split the data into a training set and a testing set, consisting of the earlier 80% and the latest 20% of the data, respectively. We use the training set to specify adequate smoothing period and the time interval for estimation of the equilibrium *A*, that is, the appropriate choices of *M* and *N* in (7) and (8), respectively.”

Applied data (line 263-272):

“We implemented the nPRM with a few choices of *M* and *N* to obtain τ-step forecasts for the training dataset of each stock index. The values of *M* range from 2 to 10 while the choice of *N* includes 30, 60, 90, 120, and 180. We set the number of forecasting steps τ = 5. We assess all combinations of *M* and *N* by the criterion of the grand mean of MAPEs across 1-step to 5-step forecasts in the training set. Afterwards, we select the combination of the best performance as the coefficient setting for prediction in the testing set. Table 2 shows that the best combination of *M* and *N* with the smallest grand mean of MAPEs in the training set for all the ten indexes. In the following, the forecasts are conducted with *M* and *N* specified for each stock index in Table 2.”

**8. In the results discussion, authors say that if applying the nPRM to the markets that are less efficient, the model coefficients may acquire more information with economic meanings and obtain more accurate forecasts than the martingale. What is the reason for this explanation? What is the definition of market efficiency? How is the author identified? If nPRM performs better in an inefficient market, why don't the authors consider using other indexes? If nPRM can perform better in other samples, I will think that this method is meaningful and contributing.**

We do not focus on the market efficiency in this work. The main contribution of this work is to present a model to characterize the stock movement with economically meaningful coefficients.

This discussion (in line 325-327,

“Despite the absence of the test in this study, the low errors of the martingale for the four Asian stock indexes imply that the markets tend to be efficient during the period of the data.”)

is a conjecture based on reference [2], which we do not present more tests (the generalized spectral martingale (EV-MDS) test or the wild bootstrap Automatic Variance Ratio (AVR) test in reference [2], for example) and empirical results in this article.

The statement (in line 331-333,

“If applying the nPRM to the markets that are less efficient, the model coefficients may acquire more information with economic meanings and obtain more accurate forecasts than the martingale.”)

could be a future work if we further research into the performance of the nPRM in markets with different levels of efficiency.

In the Introduction Section (line 17-20), we summarize research results from reference [2, 4, 5] and state

“The martingale depicts the stock movements in the strong efficient markets by the theory of random walks [4]. In contrast, many real markets are considered as the weak or the semi-strong efficient markets [2, 5]. Consequently, to forecast the prices in these markets is possible.”

Additionally, the reference [2] concludes in the article that “The EV-MDS test result shows that all the 14 return series under study are not MDS, suggesting the potential of predictability using either linear or non-linear function of past values. … Although all markets are reported earlier to be non-MDS, the AVR test shows the return series of Bangladesh, China, Hong Kong, Malaysia and Taiwan are white noise.”

We add some more examples of Asian stock indexes in this revision including the Philippines PSEi Index, Thailand SET Index, India S&P BSE SENSEX, Singapore Straits Times Index (STI), Indonesia JKSE, and Malaysia KLCI. No matter how efficient an Asian stock market in our investigation is, our proposed method nPRM can predict the stock prices with relatively low MAPE.

In details:

Applied data (line 256-260):

“The empirical study was conducted based on information from real daily closing price data of ten stock indexes from 2009 to 2019. The indexes include Taiwan TAIEX, Japan Nikkei 225, Korea KOSPI, Hong Kong Hang Seng Index, the Philippines PSEi Index, Thailand SET Index, India S&P BSE SENSEX, Singapore Straits Times Index (STI), Indonesia JKSE, and Malaysia KLCI.”

Empirical results (line 283-313)

“Figure 3 displays the 1-step forecasts of the nPRM for Hong Kong Hang Seng Index along with the market values. Figure 3 suggests that the nPRM possesses accurate forecasting ability. The patterns of the forecasts from 2-step to 5-step by the nPRM are all similar to Figure 3, and thus we omit the demonstration here. Figures 4-12 illustrate the 1-step forecasts of the nPRM for other Asian stock indexes individually, which also indicate accurate forecasts by the nPRM.”

…

“Fig 7.- Fig 12.”

“Table 3 displays the MAPEs of applying the investigated methods to all of the ten Asian Indexes from 1-step forecasts to 5-step forecasts. Since the performances of three deviation-type errors (MAPE, MAE, and RMSE) are similar, we simply report and discuss the results of the MAPEs. From Table 3, the martingale (see details in S1 Appendix) has the smallest errors followed by those from the nPRM, and the curve fitting (see details in S2 Appendix) has the worst performance.”

Empirical results (line 325-331)

“Despite the absence of the test in this study, the low errors of the martingale for these ten Asian stock indexes imply that the markets tend to be efficient during the period of the data. The martingale suggests that no information we can obtain from the past to forecast the future stock prices. However, our proposed method nPRM still captures the trend of the stock movements quite well, and provides meaningful coefficients, *A* and *k*, as well under the circumstances.”
