# Supplementary material for: Study of Asian indexes by a newly derived dynamic model
Source: PLoS One. 2022 May 2;17(5):e0266600. doi: 10.1371/journal.pone.0266600 (PMC9060367; doi:10.1371/journal.pone.0266600)
Supplement: Attachment — Submitted filename: Response to Reviewers.docx [file pone.0266600.s007.docx]

Submission No: PONE-D-21-10180R2

Dear Editor,

We greatly appreciate the reviewers’ time and effort. Your valuable comments and suggestions help us improve our paper. After careful consideration of these comments, we have addressed those comments one by one in the following responses. The original comments from the reviewer(s) are in **Black** and our responses are in **Blue**.

With Regards,

Yong Shiuan, Lee

===========================================================

**Response to Comments from Reviewer #4**

**1. At the end of the introduction, the authors list two contributions. But they are not concrete contributions from the point of view of expression. Contributions should include the innovations of this paper, please refer to expressions in other literature.**

We appreciate the reviewer’s comment. In the nPRM, the solutions of the equation were derived rigorously so that the solutions of this model can better fit the realistic stock prices of the markets than the PRM (please refer to line 135-137). A procedure is developed to process the raw stock prices without loss of low frequency of data. In summary, under the same dynamic model we proposed before, the nPRM seeks for the solutions that will not only achieve higher prediction accuracy but also provide useful economic meanings for possible applications in general practice. In addition, the nPRM can make the predictions using only raw stock prices without the need to transform them into usual economic variables such as returns or price increments. This is one of the reasons why we compared our results with two commonly used methods dealing with raw stock prices, the curve fitting and the martingale. To emphasize the contributions of this work, we revised the introduction section (please refer to line 87-98).

In detail:

Introduction (line 87-98):

“For the same dynamic model described using the differential equation in the PRM, we derive its solutions rigorously so that the solutions of the nPRM can better fit the realistic stock prices of the markets than the PRM. Two major contributions of the nPRM are as follows. Firstly, the nPRM seeks for the solutions that will not only achieve higher prediction accuracy but also provide useful economic meanings (refer to Figures 1 and 2 below). The coefficients in the nPRM, *A* and *κ*, represent the implied equilibrium price, and the convergence speed to the implied equilibrium price, respectively. Secondly, for many predictive models involved in the time series analysis, it is necessary to transform the stock prices to other economic variables, usually the returns or the price increments. The nPRM can make the predictions directly for the stock prices, which implies that the model considers all daily information in the analysis. Furthermore, the conduction of the proposed procedure is simple and convenient.”

===========================================================

**2. In the model section, the authors still haven't told me what the economic intuition of nPRM is. The authors only responded with an explanation of the variables in the model, but did not point out that it is reasonable for prices to change according to nPRM or is in line with economic meanings.**

We are grateful for the reviewer’s comment. The economic intuition is indeed important for the readers to understand the nPRM. Here, we tried our best to properly address the economic intuition of the nPRM by the phase plots shown in Figures 1 and 2. These two figures are used to demonstrate that it is reasonable for the prices to change by the nPRM for a high degree of consensus among most of the market participants. The economic meanings for the four cases of the nPRM can be explained as below. For case 1 (the 1^st^ zone in Figure 1), most market participants agree that the current price could be overestimated. Therefore, most participants expect that the future price would decrease dramatically for case 1. For case 2 (the 2^nd^ zone in Figure 1), most participants agree that the current price could be underestimated and hence expect a sharp increase in the future price. On the condition (in both case 1 and case 2), participants have high level of consensus for the price. However, the expected equilibrium price is still unknown. The coefficient *κ* is the gradient value, which represents the blow-up speed of the differential equation. It is positive for both cases 1 and 2, which indicates that the future change of the price will be fierce.

For cases 3 and 4 (the 3^rd^ and 4^th^ zone in Figure 2), most participants think that *A* is the reasonable expected price. Hence, future price will converge to *A*. This also explains why we state that the expected price *A* is the implied equilibrium price in the nPRM. The gradient value, *κ*, is negative, and its absolute value represents the speed of convergence to the expected price *A*. The existence of the implied equilibrium price also means that the market is highly efficient. The empirical study shows that the prediction error (MAPE) of the nPRM can be close to the MAPEs of the martingale, and the difference between them is less than 1%.

===========================================================

**3. In the results section, the authors selected ten stock index samples to test the explanatory power of the model. Although the ten stock markets were all market efficient during this period, the degree of market efficiency certainly varied. Therefore, the authors can compare the prediction accuracy of nPRM in terms of the degree of market efficiency. Empirical research needs a good result. If your model's prediction performance is not as good as existing methods, then you need to improve the model or limit the scope of use of the model.**

Although we do not test the market efficiency in this article (eg. the generalized spectral martingale (EV-MDS) test in reference [2]), we try to provide a glimpse of the market efficiency from sample entropy, a measure of disorder. Sample entropy quantifies the amount of regularity and the unpredictability of fluctuations in a time series. Hence, a low value of sample entropy indicates that the series is deterministic while a high value reflects randomness. We provide an extreme example in our revised manuscript to show a possible range of sample entropy. The example is a simulated series of length 180 from the standard normal distribution, namely *N*(0, 1). By repeating 1000 times, we obtain the average sample entropy as **2.21**. On the other hand, the value of sample entropy is **0**, which corresponds to a perfectly regular time series.

We add Table 5 to present the mean value of sample entropy calculated from all series used in estimation of *A* and *κ* to make forecasts dynamically in the testing set. Basically, all the mean values are close (nearly between 0.6 and 0.8), which indicates that these ten markets exhibit market efficiency of similar levels.

From the order of the mean sample entropy values, the prediction accuracy of the nPRM mostly agrees with the degree of market efficiency excep the Taiwan stock index TAIEX. TAIEX has a mean sample entropy value of 0.7052 (the second highest). The value suggests that the fluctuations of TAIEX is more unpredictable. However, the nPRM performs well in predicting TAIEX with MAPEs as 0.76, 1.00, 1.21, 1.43, 1.65 for 1-step to 5-step forecasts respectively. This means that the changes of TAIEX are still consistent with the movement that the nPRM characterizes.

The differences between the martingale and the nPRM are close, and the difference between them is less than 1%. Additionally, the coefficients in the nPRM has their own economic meanings. We will integrate the nPRM with other models (machine learning models, for example) to see if we can reduce the prediction error in the future.

In detail:

Results – Empirical results (line 325-353):

“Apart from the martingale, we can investigate the level of market efficiency by sample entropy [38, 39]. Sample entropy measures complexity. When we have a time series of length *n* as {*S*(1)*, S*(2)*,*…*, S*(*n*)} , a template vector of length *m* is defined as

, a template vector of length *m* is defined as *S_m_*(*i*) = {*S*(*i*)*, S*(*i* + 1) *,*…*,S*(*i*+*m-*1)} and the distance function *d*[*S_m_*(*i*)*, S_m_*(*j*)], *i* ≠ *j* is defined as the Chebyshev distance. Then, we define the sample entropy of the series, *SampeEn*, as

*SampEn* = − log$\frac{Bm+1}{Bm}$*,*

where *B^m^* is number of template vector pairs having *d*[*S_m_*(*i*)*, S_m_*(*j*)]≤*r*, and *r* is the tolerance.

The value of sample entropy is nonnegative. The value 0 of sample entropy indicates that the series is perfectly regular without noise while a high value reflects randomness and unpredictability. If we examine the market efficiency from the perspective of sample entropy, a high value of sample entropy indicates a market with high level of efficiency. To show how large the possible value of sample entropy from a highly random series can be, we simulate a series of length 180 from a normal distribution with zero mean and unit variance for 1000 times. The average value of sample entropy is 2.21.”

“Table 5 shows the mean value of sample entropy calculated from all series used in estimation of *A* and *κ* to make forecasts dynamically in the testing set for ten indexes in our empirical study. The mean values range between 0.5952 (Korea KOSPI) and 0.7833 (the Philippines PSEi), which represent similar complexity among these markets. We suspect that the forecasting errors should be larger for markets with higher level of efficiency since it is harder to predict the series with random noise. By the order of the mean sample entropy values and the MAPEs for the ten stock indexes, the nPRM shows a tendency toward better forecasts for series with lower market efficiency. That is, the forecasting ability of the nPRM usually increases as the mean sample entropy decreases. However, there is an exception of Taiwan TAIEX. The mean sample entropy obtained from the TAIEX series is the second highest, so that we won't expect a smaller prediction error among the ten indexes. However, the MAPEs of the nPRM for 1-step to 5-step forecasts are relatively low. The changes of TAIEX are similar to the movement which is characterized by the nPRM among the ten markets.”

===========================================================

**4. The authors proposed a newly PRM, but the comparison with the PRM was removed from the comparison of the prediction results. What is the reason? Is the comparison unsatisfactory or impossible to compare? I need a detailed comparison process.**

The details of comparison between the original PRM and the nPRM are listed below. We present the **MAPE (%)** of the original PRM and the nPRM for the testing set in the table, where the numbers of the nPRM are exactly the same as those in **Table 3** in our manuscript (page 10).

|  |  | τ = 1 | τ = 2 | τ = 3 | τ = 4 | τ = 5 |
| --- | --- | --- | --- | --- | --- | --- |
| Nikkei 225 | nPRM | 1.04 | 1.27 | 1.53 | 1.78 | 2.05 |
|  | PRM | 0.98 | 1.57 | 2.16 | 2.82 | 3.58 |
| Hang Seng | nPRM | 1.12 | 1.44 | 1.77 | 2.11 | 2.43 |
|  | PRM | 1.09 | 1.68 | 2.21 | 2.76 | 3.22 |
| TAIEX | nPRM | 0.76 | 1.00 | 1.21 | 1.43 | 1.65 |
|  | PRM | 0.79 | 1.28 | 1.75 | 2.20 | 2.68 |
| KOPSI | nPRM | 0.86 | 1.10 | 1.38 | 1.62 | 1.84 |
|  | PRM | 0.81 | 1.26 | 1.70 | 2.15 | 2.63 |
| PSEi | nPRM | 1.46 | 1.71 | 1.93 | 2.16 | 2.35 |
|  | PRM | 1.55 | 2.17 | 2.63 | 3.19 | 3.70 |
| SET | nPRM | 1.18 | 1.47 | 1.70 | 1.98 | 2.20 |
|  | PRM | 1.16 | 1.59 | 1.98 | 2.35 | 2.58 |
| BSE SENSEX | nPRM | 1.31 | 1.62 | 1.88 | 2.20 | 2.42 |
|  | PRM | 1.32 | 1.88 | 2.34 | 2.84 | 3.28 |
| STI | nPRM | 1.19 | 1.52 | 1.77 | 2.09 | 2.32 |
|  | PRM | 1.36 | 1.81 | 2.20 | 2.67 | 2.99 |
| JKSE | nPRM | 1.27 | 1.42 | 1.57 | 1.73 | 1.84 |
|  | PRM | 1.27 | 1.73 | 2.14 | 2.49 | 2.80 |
| KLCI | nPRM | 0.98 | 1.30 | 1.58 | 1.89 | 2.07 |
|  | PRM | 1.07 | 1.50 | 1.93 | 2.44 | 2.80 |

This table shows a similar phenomenon that the PRM has a tendency toward higher prediction error for multiple step forecasts. Although some (4 out of 10) of the MAPEs of 1-step forecast by the PRM are smaller than those by the nPRM, it simply happens by chance when the cumulated error is still small. The results of the above Table are not significant on the conclusions of this study.

The comparison has been made in the manuscript of our previous version. Here, we quote some sentences as follows.

“Comparing the nPRM with the original PRM, although the PRM obtains smaller 1-step forecast errors, the PRM obtains larger errors than the nPRM with the increase of the number of forecasting steps.”

“The original PRM has some flaw in the integration process and does not require smoothing for the stock price. These disadvantages cause errors in forecasts. The errors are small when the time interval of forecast is short, but they grow with the increase of the time length. The newly proposed method nPRM improves these weaknesses. Therefore, the forecasting errors are notably smaller than the PRM for multiple step forecasts.”

We removed this comparison due to limited space. It’s fair to compare the proposed method with other commonly used models instead of a method we proposed previously, which is the more general way to demonstrate the empirical study.
